# Whole Exome Sequencing in South Africa: Stakeholder Views on Return of Individual Research Results and Incidental Findings

**DOI:** 10.3389/fgene.2022.864822

**Published:** 2022-06-08

**Authors:** Nicole Van Der Merwe, Raj Ramesar, Jantina De Vries

**Affiliations:** ^1^ UCT/MRC Genomic and Precision Medicine Research Unit, Division of Human Genetics, Institute for Infectious Diseases and Molecular Medicine, Department of Pathology, Faculty of Medicine and Health Sciences, University of Cape Town, Cape Town, South Africa; ^2^ Department of Pathology, Faculty of Medicine and Health Sciences, Stellenbosch University, Tygerberg, South Africa; ^3^ Department of Medicine, Faculty of Health Sciences, University of Cape Town, Cape Town, South Africa; ^4^ Neuroscience Institute, Faculty of Health Sciences, University of Cape Town, Observatory, South Africa

**Keywords:** whole exome sequencing, incidental findings, secondary findings, return of results, genetic counselling, South Africa

## Abstract

The use of whole exome sequencing (WES) in medical research is increasing in South Africa (SA), raising important questions about whether and which individual genetic research results, particularly incidental findings, should be returned to patients. Whilst some commentaries and opinions related to the topic have been published in SA, there is no qualitative data on the views of professional stakeholders on this topic. Seventeen participants including clinicians, genomics researchers, and genetic counsellors (GCs) were recruited from the Western Cape in SA. Semi-structured interviews were conducted, and the transcripts analysed using the framework approach for data analysis. Current roadblocks for the clinical adoption of WES in SA include a lack of standardised guidelines; complexities relating to variant interpretation due to lack of functional studies and underrepresentation of people of African ancestry in the reference genome, population and variant databases; lack of resources and skilled personnel for variant confirmation and follow-up. Suggestions to overcome these barriers include obtaining funding and buy-in from the private and public sectors and medical insurance companies; the generation of a locally relevant reference genome; training of health professionals in the field of genomics and bioinformatics; and multidisciplinary collaboration. Participants emphasised the importance of upscaling the accessibility to and training of GCs, as well as upskilling of clinicians and genetic nurses for return of genetic data in collaboration with GCs and medical geneticists. Future research could focus on exploring the development of stakeholder partnerships for increased access to trained specialists as well as community engagement and education, alongside the development of guidelines for result disclosure.

## Introduction

The past two decades have seen a considerable increase in the use of genomic methods in health research and clinical care. Studies have assessed the use of whole exome sequencing (WES) for a broad spectrum of disorders towards increasing the diagnostic yield in patient populations restricted to specific phenotypes, to up to 80% (Neveling et al., 2013; Yadava and Ashkinadze, 2017). In South Africa (SA), WES is currently used only in the research setting at approximately 10 facilities/institutions, few of whom have published exome data (Roberts et al., 2016; van der Merwe, 2016; Pierrache et al., 2017; Baynam et al., 2020; Sawe et al., 2020). An advantage of WES over individual gene or next generation sequencing (NGS) panel testing is that the stored data enables re-analysis of new as well as pharmacogenomic genes that are linked to the patient’s phenotype. WES is furthermore reported to be beneficial in patients with atypical presentations as it may reduce the time to diagnosis as well as the psychological and financial burdens associated with prolonged investigation (Monroe et al., 2016; Harris et al., 2017; Stark et al., 2017; Walsh et al., 2017).

Although WES has great potential to improve diagnostic accuracy, health outcomes and resource utilization, several limitations contribute to a widening gap between the generation of complex genetic data and its use in daily clinical practice. Return of individual WES results, notably unanticipated or incidental findings (IFs) generated in the research setting in particular, remains a complex and highly debated issue (Wolf et al., 2008; Berg et al., 2011; Bredenoord et al., 2011; Ortiz-Osorno et al., 2015). One of the key challenges relates to what to do with unanticipated individual research results. Internationally, various recommendations on how to deal with findings that are outside of the test indication [secondary findings (SFs)], and guidance for their return, have been established (ACMG policy statement, 2021). However, in Africa, no standardised guidelines exist. While the H3Africa consortium (https://h3africa.org/) has developed a policy for the return of findings, little empirical data exists about the views of African professional stakeholders on the topic. The Individual Findings in Genomics Research (IFGeneRA) project conducted under the umbrella of H3Africa has established a working group tasked to generate and publish an African-specific list of reportable genes/variants (Wonkam & De Vries, 2020). These authors proposed that the African genetics community in collaboration with international experts, generate three priority lists. Until such guidelines are developed and publically available, we refer to IFs.

To date, this is the first South African study conducted to map the experiences of health professionals regarding the practice of WES and return of IFs. Mwaka et al. (2021) conducted a qualitative study on Ugandan researchers’ perspectives on return of individual genetic findings to research participants. They asserted that community engagement, reconsenting and adequate preparation of participants to safely receive individual results, may be achieved by building capacity and increasing access to clinical genetics and genetic counselling (GC). Given the possibility of future reinterpretation of data, non-disclosure of actionable research results may be considered unethical (Bombard et al., 2019), however, the no-return criterion may apply when results from genomic studies are intended as generalisable findings instead of those demonstrating clinical utility (AESA, 2020 policy paper, 2020). Several studies conducted in high income countries that have examined the expectations of research participants and attitudes toward the return of individual research results with potential clinical significance, have shown that a majority of individuals want genetic data returned to them (Christenhusz et al., 2013; Wolf, 2013). In addition, some studies revealed that many research participants want all these data regardless of its “actionability” or clinical significance (Bollinger et al., 2013; Harris et al., 2013; Mackley et al., 2017). Alternatively, the right of participants (not) to know has also been extensively discussed (Herring and Foster, 2012). The distinction between research and clinical sequencing has served as a justification for not returning IFs unless they meet the criteria of clinical utility, actionability, and clinical urgency (Clift et al., 2015).

Limited work has been done to investigate obligations to return IFs in lower-and middle income countries, including those in Africa (Kerasidou, 2015). The obligation of healthcare professionals to patients - to improve patients’ health, avoid psychological and social harms and provide prolonged disease-free survival - should arguably extend to the return of any findings which would facilitate the latter, even in the African setting where the healthcare system is limited and heavily under-resourced. In the local setting, pertinent issues that affect WES are further muddled by the challenges inherent to the public healthcare system i.e. the lack of infrastructure and adequately trained personnel, high burden of communicable disease and high incidence of poverty experienced by patients (Mayosi et al., 2012; Barron and Padarath, 2017; Manyisa and Van Aswegen, 2017; Bradshaw et al., 2019). Another important challenge hindering the return of IFs is insufficient validation of genomic findings for genetically diverse African populations. Most of the currently well-established bioinformatics tools and variant calling pipelines are benchmarked using non-African genomic data, and available reference genomes are biased toward non-African populations, resulting in mislabelling of rare variants and potential misclassification by in silico prediction tools (Bao et al., 2014; Martin et al., 2018; Bope et al., 2019; Wonkam and De Vries, 2020).

Against this backdrop, this study aims to explore the experiences and perspectives of pertinent stakeholders on the return of IFs in the South African research context. Specifically, we conducted a study with clinicians, genomics researchers, and genetic counsellors (GCs) who are involved with WES in the research and/or clinical setting.

## Materials and Methods

Essential aspects of the study context and methods, research team, findings, analysis and interpretations are reported according to the 32-point consolidated criteria for reporting qualitative research (COREQ) checklist (Tong et al., 2007).

### Study Design

This study was designed as an exploratory qualitative study which enables exploration of the essential qualities of complex phenomena. Phenomenology is a qualitative research method that involves a detailed examination of participants’ perceptions of their shared and lived experiences (Smith and Firth, 2011).

### Research Team

This research was conducted to fulfil the requirements for an MSc (Med) Genetic Counsellor degree at the University of Cape Town, SA. NvdM and JdV are female, RR is male. Whilst being an intern Genetic Counsellor at the time, NvdM had also obtained a PhD degree in Pathology from Stellenbosch University. RR is a Genetic Counsellor and Professor in Human Genetics; JdV is an Associate Professor in Bioethics with a background in Sociology. NvdM obtained training in qualitative research methods as part of her genetic counselling degree; she also received guidance from JdV throughout the process. As a scientist with experience in WES variant interpretation and reporting, NvdM had a tendency to focus on the technical rather than the ethical aspects of WES during the interviews. JdV and NvdM worked together to minimise such bias and ensure that interviews covered broader aspects of WES beyond the technical ones only.

### Participants

A purposive sampling method was used to select participants most likely to have the expertise or experience to provide valuable insights on the research topic. Some participants were known to members of the research team and were recruited from the University of Cape Town, Stellenbosch University, Red Cross War Memorial Children’s Hospital as well as two private medical facilities from September 2016 to March 2017. Pertinent stakeholders such as clinicians, genomics researchers and GCs involved in any stage of the WES process, both in the clinical and research settings, were included in the study. Participants were selected based on their (presumed) insight relating to the return of individual WES results. Stakeholders were invited to participate in this study by means of an email that included a description of the research to be conducted, the ethics approval document and consent form. While twenty-eight participants were invited to participate, 17 were recruited as part of the study. Interview dates and times were subsequently established with those who responded, and face-to-face, semi-structured interviews conducted at their offices/consultation rooms. Interviews were conducted by NvdM with no-one else present. All interviews took place between October 2016 and April 2017, were conducted in English and lasted between 45 minutes and an hour. Saturation was reached by the 14th interview and an additional three interviews were conducted to ensure this.

### Instrumentation and Procedures

The topic guide was designed based on the literature, including theoretical work that identifies and describes issues pertaining to WES/return of findings, as well as empirical studies conducted elsewhere in the world. Questions were carefully framed to remove bias and any suggestive wording, and to ensure neutrality. They related to stakeholder experiences and understanding of various aspects pertaining to WES; examples of its use; the role of WES in clinical practice; consent relating to return of findings; return of WES results and data storage and reuse. Finally, the topic guide was reviewed by the two project supervisors and piloted twice to elucidate potential weaknesses, flaws or limitations in its design. This enabled adaptations to be made to the topic guide prior to implementation of the study. During interviews, closed-ended questions were used to capture demographic data, followed by a series of open-ended questions using the topic guide. Interviews were audio-recorded and no repeat interviews were conducted.

### Data Analysis

The framework approach was selected to analyse the data as it allows researchers to trace, map and categorise the perspectives of individual participants across themes or other key attributes. This approach was initially developed in the 1980’s for social policy research and is a useful tool for qualitative data analysis in the healthcare setting (Smith and Firth, 2011). A systematic, four-step process was used to facilitate rigorous data analysis and transparent data management, ultimately resulting in the categorisation of data into themes and interrelated sub-themes (Spencer et al., 2003).

The interview recordings were transcribed verbatim by the researcher (NvdM). An alphanumeric code (e.g., P1-P17) was assigned to each participant to ensure participant confidentiality and any identifiable information removed during transcription. This was followed by randomisation of this code to disrupt the sequential order of interviews. Transcripts were not returned to participants for verification. They were initially open-coded to generate an open-coding scheme, which was subsequently discussed with supervisors to develop a hierarchical coding scheme. This was followed by importing transcripts into a data management software program called NVivo11 (QSR International). The entire dataset was coded and analysed using the Framework Table function in NVivo. Initial steps of this analysis occurred simultaneously with data collection. Field-notes were used to facilitate interpretation of the data and were subsequently discussed with the research team.

## Results

A total of 17 participants were interviewed in this study (Table 1). They had various levels of experience with WES, being either directly or indirectly involved in a WES process in the research, or clinical, or both settings (Table 2). “Direct” involvement refers to direct interaction with WES data or the patients whom WES was performed in, as opposed to “indirect” involvement where the use of WES is limited to the environment the participant operates in. The themes and sub-themes identified by transcript analysis, as described in the methods section, are summarised in Table 3. For the purpose of this study, Only extracts of the data pertaining to the themes “WES practice” and “incidental findings”, are presented in this paper.

**TABLE 1:** Themes and sub-themes generated during the data analysis phase of the study.

Themes	Sub-themes
Whole exome sequencing (WES) practice	WES selection criteria
	WES approach
	Barriers and opportunities
Incidental findings in WES	Knowledge and experience
	Type of results that should be returned
	Result validation
	Community engagement
Ethical considerations and implications	Risks and benefits
	Consent models
	Type of information that should be provided
	Data ownership, accessibility and storage
	Local guidelines: views and recommendations
Return of findings	Who should return findings
	The role of the genetic counsellor
	The need for training in the field of genomics

**TABLE 2:** Demographics of the study participants.

Participant code	Years of practice in field: 1) 1–5; 2) 6–10; 3) 11–15; 4) 16–20; 5) >21	Sector: private practice (PP)/public institution (PI)	Formal training in genetics (yes/no)	Direct/indirect use of WES	Setting (research, clinical or both)
P1	4	PI	Yes	Direct	Research
P2	2	PI	Yes	Direct	Both
P3	2	PI	Yes	Direct	Research
P4	5	PP	No	Indirect	Research
P5	3	PI	Yes	Direct	Research
P6	2	PI	Yes	Direct	Research
P7	5	PI & PP	No	Indirect	Research
P8	4	PP	No	Indirect	Research
P9	5	PI & PP	Yes	Direct	Research
P10	2	PI	Yes	Direct	Research
P11	2	PI & PP	Yes	Direct	Both
P12	5	PI	No	Direct	Research
P13	5	PI	No	Direct	Both
P14	3	PI	Yes	Direct	Research
P15	3	PI	Yes	Direct	Research
P16	3	PI	Yes	Direct	Research
P17	2	PI	Yes	Indirect	Research

**TABLE 3:** Number and proportion of study participants, listed by profession.

Stakeholder		Participants	
		N = 17	% of total
Clinician	Other	5	29
	Medical Geneticist	3	18
	Genetic Counselor	3	18
Researcher	Medical Scientist	6	35

### Participants’ Understanding of Various Aspects Pertaining to Whole Exome Sequencing

Participants displayed various levels of understanding of WES technology, with genomics researchers articulating a greater depth of knowledge and understanding of the technical scope and limitations of WES compared to clinicians. When discussing how and when WES is used in clinical practice and health research, most participants who were directly involved in WES asserted that the selection of patients is based on the complexity or heterogeneity of the disease and the lack of findings using standard genetic testing approaches. Other interviewees, particularly those in the research setting, based the selection of patients for WES on the occurrence of treatment failure, novelty or “publishability” of the condition. With regard to the sequencing approach, many interviewees in both settings described that their first port of call would be to use a NGS or a virtual WES panel, and only to turn to full exome sequencing analysis when these options have been exhausted. A virtual gene panel refers to restricted analysis of disease-associated genes sequenced using WES. The reasons for using targeted gene panels were ascribed to the fact that it narrows the spectrum of findings (both WES and NGS panels) and increases the sequencing coverage (NGS panels).

“Targeted WES seems to be, from a clinical point of view, perhaps a more viable option, because then if you have at least some narrowing down of what you are looking for, the ability to perhaps target certain genes seems to be useful. So, we have found that successful in a group of patients…” - P7

“…there’s two very important reasons to pursue this test (WES) and the one is, if there’s treatment failure and secondly, if there’s a family history that cannot be accounted for by the initial BRCA screening, whether it’s the founder mutations in a high risk group or a full BRCA1 or 2 screen.” – P9

Some participants ascribed their preference of a virtual panel to the lower cost of WES, compared to constructing/using a multi-gene NGS panel. Other interviewees described their point of departure to be full WES analysis, to avoid preclusion of novel findings.

While various challenges have been described in the literature with regard to the technical and ethical aspects of WES, interviewees described that specific barriers such as resource scarcity (including finances, skilled professionals and population-specific genetic knowledge) and lack of infrastructure are pronounced in the South African setting and impede the routine use of WES.

“Resources, definitely. Human resources, mainly. As I understand it, the biggest limiting factor is, in South Africa, well, one thing is the data storage, apparently, and the other thing is the bioinformatics, the manpower … obviously being able to that right. From our experience here, it does also sound like there has been issues with that, in that two labs both doing WES, are coming up with different things.” - P6

“…when we do have the policies and the data management capacity in place, the bioinformatics support in place - which hopefully will happen but I am not holding my breath because that would mean getting a significant increase in staffing and funding - then we can start thinking about opening up the scope that we offer [returning IFs/SFs].” – P2

An array of opportunities were identified by interviewees to overcome such barriers, including the generation of a knowledge database that serves as a source of genetic information pertaining to local, indigenous populations. In addition, multidisciplinary collaboration and training of health professionals in the field of genomics were suggested as a means of understanding the incorporation of genomic data into clinical practice.

*“*If you are working out of European population (databases), you are going to find a lot of novel variants. So, if we have to implement it in the clinical practice, we need to enrich first of all the exome databases, and the reference example from all the populations. Otherwise you are going to have a misrepresentation if you use the exome database that is enriched just for the population of European descent, which is the case now.” – P14

### Participant Views on What Findings Should Be Returned

More than 80% of stakeholders had not themselves encountered IFs, since they had wilfully avoided the possibility of obtaining them by using more targeted approaches.

“…if you’re not using virtual panels, or you’re not just looking at the genes of interest, in that way you would complicate your life.” – P6

“…I would never ever look at everything, simply because I do not want to know. That could point to incidental findings. […] So, you cannot just go and open up this can of worms if you are not ethically prepared for it. And you need to have policies in place to say, if we find something, what are we going to do with it. …If we have the capacity and we have the infrastructure and the policies in place then we can start thinking about opening up the scope that we offer. […] I don’t even want to touch it at the moment and I avoid it at all costs.” – P2

A range of views were expressed with regards to the kind of WES results that should be returned, echoing the five principles related to actionability as well as the availability of resources to return such results. Considerations were given to the likely clinical and psychosocial impact of the research result, and the autonomy of the patient. Generally, participants expressed that there is an obligation to return any results with life-saving potential or those that will impact on the clinical management of the patient in terms of prevention, surveillance and medical intervention - whether obtained in the research or the clinical setting. Approximately 33% of interviewees (mostly researchers) described an obligation to return results pertaining only to the disease that is being studied, while mainly “other” clinicians advocated return of any actionable results, including those outside of the disease in question. Overall, medical scientists seemed to be most unfavourably inclined to returning IFs, compared to clinicians who responded more favourably in this regard.

“I believe that feeding back of important results to families is the only thing. Whether we do it the same way they do it in America; the answer is: definitively not. […] Important for me would be a life-threatening expectation given in an individual, and for which medical care can provide some therapeutic solutions, or at least some follow-up and some preventive solution. I believe that the [56] actionable genes are a good start. We need to define, according to the context, which type of results will have to be fed back.” – P14

Participants made a distinction between the clinical and research setting in relation to returning IFs. Some believe that in the clinical setting, there is an obligation to disclose any IF that points to a disease or the predisposition thereof, particularly due to the legal implications associated with non-disclosure – whether actionable or not. Lastly, some participants held that there should exist two categories of results for disclosure: a standardised list for all participants as well as a patient-specific list that is to be informed by the personal, medical and family history of the patient. Two participants contended that the ACMG’s list of reportable SFs is a good point of departure, but that eliciting the view of the general population in terms of what they think should be returned, is of utmost importance.

“…I believe that the [now 73] actionable genes are a good start. We need to define, according to the context, which type of result will have to be fed back. […] But before we do that, it will be always nice to know what the people think about that: what does the stakeholder, what does the general population think about that? So, I do think that there is a fair amount of qualitative type of research to be performed in the population to know, first of all, their knowledge of genetics, and second, their knowledge of all this new emerging technology, and their willingness eventually to have those results back. […] We need to know what our people think first.” – P14

### Next Generation Sequencing Result Validation

Study participants interpreted the concept of result validation as confirming that the variant detected by WES is actually present (exclusion of false positives), as well as confirming the clinical significance or pathogenicity of the variant (disease-causing potential). While 90% of participants thought that it is crucial to confirm WES results with Sanger sequencing, some were of the opinion that it is merely necessary to confirm results that were obtained in an unaccredited setting and/or those that will be acted upon.

“I would never send out a result if it hasn’t been confirmed with Sanger sequencing at all…” – P2,

“I think that it’s just probably not practical to Sanger sequence every single variant. But I do think if one is doing something - in a not carefully quality-controlled environment - that is going to have a healthcare intervention, you should probably double check that result before you act on it.” – P16

While some participants contended that results should be confirmed strictly in an accredited environment, others believe that the lack of accredited laboratories in (South) Africa needs to be taken into account.

“Validation on sanger sequencing should be done in a diagnostic kind of environment as far as possible.” – P5

“The gold standard of diagnostic validation is something that has been set in the Western context. But if we have the context of the African continent, we have so few labs that can actually do molecular diagnostics - and I will be fairly confident that even an unaccredited lab that is working in a reasonable environment, clinical environment, can be trustworthy from the beginning. We have to have a two-stage approach. One stage is that we work with the resources that we have, and in the second stage, we can move forward to our own type of system of validation. But the fact that we cannot validate should not stop us from feeding back that result to the patient.” – P14

## Discussion

Recent years have seen a local increase in the use of WES for the generation of genetic reference data from historically marginalized populations to help distinguish real from spurious findings and to improve the diagnosis of rare diseases with a low diagnostic rate including primary immunodeficiency, retinal degenerative (Roberts et al., 2016; Pierrache et al., 2017) and mitochondrial disorders, neurodevelopmental conditions, hearing loss (Walsh et al., 2017; Wonkam et al., 2021), and cancers (van der Merwe et al., 2017; Sawe et al., 2020). In the breast cancer setting, van der Merwe et al. (2017) and Sawe et al. (2020) described the use of WES with appropriate consent for simultaneous assessment of inherited-, lifestyle- and therapy-induced risk, toward improved patient management across the continuum of care. While WES and whole genome sequencing (WGS) (Glanzmann et al., 2021) is still only used in research, NGS panels have recently been adopted in the clinical setting, particularly for the diagnosis of cancers. Less than 40 molecular biology medical laboratories across SA including some of the centres from which participants were recruited, are accredited with the South African National Accreditation System (https://www.sanas.co.za/), and very few laboratories’ NGS gene panels are currently accredited for diagnostic use. Interviewees, and particularly the researchers, seemed to be well versed in the benefits, risks and limitations of WES and were optimistic about its future implementation in clinical practice.

An interesting observation was that in the absence of local policies on how to manage IFs, stakeholders have largely avoided obtaining them. The majority of participants expressed a preference for targeted approaches such as the use of disease-specific NGS panels, for the purpose of reducing the likelihood of IFs and variants of uncertain significance (VUSes). This is in line with previous recommendations of the American Society of Human Genetics (ASHG) to use targeted sequencing or selective sequence analysis, to minimise the likelihood of discovering IFs particularly during adolescent testing (Botkin et al., 2015). Conversely, ACMG policy statements are largely in favour of disclosing SFs in clinical exome and genome sequencing regardless of patient age. The ACMG furthermore has not explicitly considered reporting of SFs with NGS or WES virtual panel testing until 2019/2020, when they described various challenges related to obtaining consent and the additional workload incurred when using these test methods (Miller et al., 2021).

The development of African guidelines for disclosure of genomic findings - and in particular IFs - are imperative given the novelty and high level of variation of African DNA, and the impact of its absence on local implementation of WES. Due to lower costs, customisable testing options and confidence in laboratory processes, a number of stakeholders are routinely making use of overseas-based sequencing (NGS) facilities. This phenomenon has been somewhat criticised due to the embargo and otherwise unlimited access of African data to non-African scientists, but not opposed in lieu of the lack of standards and limited clinical implementation of this technology in SA. The lack of guidelines for IFs and the complexities relating to variant interpretation poses some of the main challenges that are impeding the widespread adoption of WES.

On a technical level, low coverage of certain genomic regions, the discordance between variant-calling pipelines and bioinformatics tools, and biased reference genomes used in WES further obstructs its routine use in the clinical setting. Genetic heterogeneity is a major limitation when dealing with African genomic data as it may increase the number of false positive and false negative findings in relation to the reference genome used. When working with an ill-representative reference genome, the increased amount of rare (especially missense) variation detected with lack of corresponding functional information makes it difficult to establish pathogenicity. Equally, if the depth of germline sequencing is too low, false positive variation due to systematic errors cannot be distinguished from the presence of somatic variation. Several studies evaluated variant filtering tools and criteria for categorising novel variants, reviewed available databases and in-silico prediction tools, and proposed recommendations for analysing variation in African genomes (Kessler et al., 2016; Bope et al., 2019). Variant annotation is furthermore a crucial step in the analysis of NGS data and results can have a strong influence on variant classification. As may be the case when working with African genomic data, incorrect or incomplete annotations may cause researchers to miss, overlook or dilute note-worthy DNA variants in a pool of false positives (McCarthy, et al., 2014). Since less than 2% of human genomes analysed comprises that of African individuals (Sirugo et al., 2019), variant annotation is expected to improve as the availability of African genomic data increases, with subsequent increase in classification accuracy. One of the study participants reported that ∼70% of causative variants detected using WES in African patients were novel and not found in publically available databases. They emphasised the need for exome databases and the reference genome to be enriched for African data in order for WES to be successfully implemented in clinical practice. Approximately 70% of the current human reference genome, GRCh38, is derived from a single individual and therefore it fails to capture the genetic diversity of most populations (Ballouz et al., 2019). Methods proposed to overcome this issue include nucleotide additions and extensions to GRCh38, graph-based references that simultaneously represent multiple, diverse populations and the generation of population-specific consensus sequences *via* *de novo* assembly of raw read data (Huang et al., 2013; Duan et al., 2019; Li et al., 2020). Population-specific reference genomes/panels developed to date include European, East Asian and African (Yoruban) major allele reference sequences Dewey et al. (2011), Vietnamese (Nguyen et al., 2015), Danish (Maretty et al., 2017), Chinese (Zhang et al., 2021), Japanese (Takayama et al., 2021) and Arab genome sequences (Fakhro et al., 2016; Elbait et al., 2021), and an African American reference panel (O’Connell et al., 2021). In 2021, Ebert et al. (2021) published a new, more comprehensive reference dataset reflecting 64 assembled human genomes representing 25 different human populations from across the globe. In a Nature commentary, Wonkam (2021) shared the vision of H3Africa to sequence three million African genomes (3MAG) in order to build a more representative reference genome. van der Merwe et al. (2017) previously compared the GRCh37 (hg19) reference genome to a European major allele reference sequence during WES in breast cancer patients and controls. The authors demonstrated that using an ethnically concordant reference genome increases the specificity and sensitivity of WES results.

WES is furthermore susceptible to multiple source errors including sequencing errors, incorrect mapping (“mismapping”), and random sampling (Kiezun et al., 2012). In light of these caveats, WES data is routinely validated with Sanger sequencing which is considered to be the gold standard for result confirmation (McCourt et al., 2013). According to the study participants, three aspects pertaining to the validation of WES data are important to consider: whether and what results should in fact be confirmed using Sanger sequencing; whether validation should occur in an accredited laboratory; and whether or not this depends on the setting. Nearly 90% of participants expressed that it is crucial to confirm all reported WES results with Sanger sequencing (or PCR), especially if the result is to be used for family screening or prenatal testing. However, not all of these stakeholders contended that variant confirmation should be done in an accredited laboratory. The rest were of the opinion that the technology has improved to the extent that we may be able to trust it, and that only findings obtained in an unaccredited environment that lead to a healthcare intervention, should be confirmed. It appears that even in the literature there is no absolute consensus regarding the need for NGS variant confirmation using Sanger sequencing. While some studies highlight the importance of Sanger confirmation, others do not (Strom et al., 2014; Beck et al., 2016; Mu et al., 2016).

Interviewees emphasised the need for context-specific practices, i.e., practices that take into consideration limited local capacity and the lack of accredited laboratories in the (South) African setting. While some participants held that validation should be done exclusively in an accredited facility, others contended that they are fairly confident that results generated in an unaccredited laboratory operating in a reasonable clinical environment, may be trustworthy, and that the lack of accreditation should not hamper or obstruct the return of results to patients.

Additional challenges mentioned by stakeholders that are echoed in the literature (Bertier et al., 2016) include the lack of data management, storage and bioinformatics capacity, funding and our understanding of gene-gene/gene-environment interactions and protective effects. An array of opportunities were identified to overcome the barriers to widespread adoption of WES in clinical practice. These include: obtaining buy-in from the private sector, government and medical insurance companies for funding tests, and the generation of a locally relevant reference genome or knowledge database. The ability to perform exome sequencing and functional studies in parallel would furthermore aid interpretation of rare/population-specific missense variation if accompanied by family and case-control studies. In addition to this, multidisciplinary collaboration and training of health professionals in the field of genomics were suggested as a means of facilitating better understanding, interpretation and incorporation of genomic data into clinical care. This is corroborated by the literature, which suggests that variant interpretation requires the collaborative intervention of different highly-trained specialists (Newman and Black, 2014) including bioinfomaticians, biologists and clinicians (Sisodiya, 2015). Krause (2019) highlighted the requirements for an improved genetic testing service in SA. These include correct indications for testing (appropriate selection criteria), the generation of a local database resource, implementation of state-of-the-art testing based on research, and increased academic training including active training of GCs and medical geneticists.

### What Findings Should be Returned?

IFs obtained with WES is a contentious subject (Bredenoord et al., 2011; Wolf, 2013). While a limited number of interviewees preempted IFs alongside extension of their WES analyses, most participants did not foresee obtaining IFs, largely because they confine analysis and return of findings to more targeted approaches. In addition to avoiding IFs due to the lack of standards for dealing with them, concerns regarding available infrastructure and capacity for managing them also arose. Specifically, stakeholders emphasized that the lack of resources for returning primary findings is exaggerated by the prospect of delivering an even greater volume of (unanticipated) results with potential clinical importance. This larger volume and wider scope of results calls for an increase in several resources, which would place additional strain on an already-stretched healthcare system. While an average of 30% of patients who undergo diagnostic WES obtain a molecular diagnosis (Valencia et al., 2015; Trujillano et al., 2017), ∼3% of individuals are also left with an IF of unclear significance or secondary genomic finding (Yang et al., 2014; eMERGE Clinical Annotation Working Group, 2020). VUSes account for 40% of variants discovered to date (Federici and Soddu, 2020). WES of 6503 individuals of European and African ancestry have identified actionable variants in 2% and 1.1% in these groups, respectively (Amendola et al., 2015). The uniqueness and missing heritability of African populations raised the question of whether we should use existing recommendations developed by the ACMG or 100,000 Genomes Project (www.genomicsengland.co.uk) and customise these to the local context, or develop African-specific actionable targets prior to the implementation of local policies (Wonkam and De Vries, 2020). As local institutions are expanding WES efforts, determining the relevance and application of international recommendations across diverse populations have become paramount. Currently, the IFGeneRA project involving both local and international experts in the field, is developing African-specific guidelines for returning genomic findings obtained using African DNA (Wonkam and De Vries, 2020) (https://h3africa.org/). This is a significant endeavour since ethnicity is likely to be an important predictor of penetrance of pathogenic variants (Trinh et al., 2014). Stakeholders contended that penetrance studies require the analyses of diverse populations, since allelic background is likely to influence the phenotypic consequences of IFs.

In relation to the type of WES results that ought to be reported, a range of views were expressed by stakeholders. Although varied in terms of the setting in which WES results are obtained, views were largely grounded in the clinical and psychosocial impact of the research result, and the autonomy of the patient. Participants expressed that in both the clinical and research settings, there is an obligation to deliver results with life-saving potential or those that will impact the clinical management of the patient in relation to prevention, surveillance or medical intervention - if related to the disease in question (or test requested). The literature largely supports the disclosure of variants taking into consideration actionability, pathogenicity, phenotypic severity, analytic validity, and participant consent (Fabsitz et al., 2010; Cassa et al., 2012). While stakeholders agreed upon the criteria of actionability, 33% of participants (mainly researchers) contended that only pertinent research results should be delivered, while clinicians (other than GCs and medical geneticists) expressed that any actionable results, whether primary or incidental, should be returned. The literature supports the latter (Clift et al., 2015). Interestingly, among stakeholders, the clinicians appeared to be more focused on the potential clinical impact of IFs and their duty to return meaningful results whereas scientists were more focused on the lack of guidelines and limited resources available for seeking, confirming and reporting IFs. This disparity potentially relates to the differential allocation and/or availability of resources within the two settings as well as to the ACMG recommendations which states that clinicians have an obligation to deliver IFs that pertain to their patients’ health and management (American College of Medical Genetics and Genomics Board of directors, 2021). Hallowell et al. (2015) argued that issues concerning the return of IFs are influenced by the context in which WES is performed, namely the clinical or research setting, and that decisions about the disclosure of IFs generated in the clinical context are much less ethically contentious than those detected in the research setting.

Five principles for determining whether research results should be returned to participants were outlined in the literature and reflected in this study, including obtaining appropriately informed consent, analytical validity of results, the possibility of result-based intervention and adequate mechanisms and resources in place to return results (McGuire et al., 2008; Fabsitz et al., 2010; Munung et al., 2016). GCs may be in the ideal position to proactively define their roles in the WES process. These may include an essential role in the consenting process where they are able to obtain informed consent in a staged manner through ongoing patient interaction, thereby potentially mitigating some of the concerns related to the high volumes of information to be relayed and the potential negative emotions they may provoke. Ormond (2013) discussed several areas in which practice will likely change as we move from genetic to “genomic” counselling, while Patch and Middleton (2018) outlined its current implementation in GC training curricula. The progressive adoption of WES into the clinic furthermore allows for the awareness, education and training of various stakeholders (Brazas and Ouellette, 2016). In (South) Africa where the number of GCs and medical geneticists are few, we contend that laboratories, researchers, public and private institutions housing clinicians, genetic nurses and other healthcare workers ought to work in partnership with GCs to disseminate genetic information (Baynam et al., 2020). The establishment of an online genetic counselling platform (FamGen Counselling™) to facilitate nation and ultimately continent-wide access through multidisciplinary collaboration and integrated GC networks, was an important consideration towards reducing the widening gap between increased amounts of genomic data being produced and insufficient access to/underutilisation of trained genetics specialists who are able to make sense of it. Efforts are furthermore geared towards upscaling and certifying the conventional and unconventional GC workforce, respectively, given the pace at which genomics is incorporated into clinical practice. To furthermore promote multidisciplinary stakeholder training and interaction, various online courses and webinars attended by local and international genomics researchers, clinicians and GCs are periodically conducted to share and identify gaps in current knowledge and address relevant clinical questions encountered in oncogenomic practice. Although knowing which reportable findings stakeholders deem important, is essential for developing local guidelines, a researcher and clinician highlighted the need to determine the views of the general population in this regard. To achieve this, a fair amount of qualitative, community or patient-focused research is required.

## Strengths and Limitations of the Study

This study is the first of its kind to employ qualitative methodology to explore stakeholder views and experiences of WES IFs in the South African setting. It highlights the barriers related to the local context but also identifies valuable opportunities to overcome these barriers – which may be relevant to other lower and middle-income countries. As there are no other qualitative research regarding the role of GCs in returning WES findings, this research is valuable for the growing field of GC in SA.

Being the researcher’s first introduction to qualitative research methodology, it is possible that the quality of the interviews has improved over the course of the study. Furthermore, purposive sampling may have lent itself to ascertainment bias. Lastly, the views of other health professionals who are not involved in WES, patients and the community as a whole, were not included in this study.

In conclusion, this study provides valuable insights into the views and experiences of local stakeholders involved in the WES process in SA, which have not previously been explored. Appraisal of stakeholder experiences and practices facilitated the understanding the current operations, challenges and barriers within respective settings, but also identified opportunities and potential solutions which may ultimately inform the adoption of WES into clinical practice. Current roadblocks that impede this process include a lack of local guidelines for returning IFs; complexities related to variant interpretation due to lack of functional studies and underrepresentation of people of African ancestry in the reference genome, population and variant databases; the lack of resources both in terms of skilled personnel and infrastructure for variant confirmation and follow-up. The demand for adequately skilled professionals will likely be met in two ways: the upskilling of health professionals in the field of genomics and the upscaling of GC use and accessibility to a larger volume of the (South) African population.

## Data Availability

The data supporting the findings and conclusions of this article will be made available by the authors without undue reservation.
